# Suicide as slow death: towards a haunted sociology of suicide

**DOI:** 10.1177/00380261231212764

**Published:** 2023-11-29

**Authors:** Amy Chandler, Sarah Wright

**Affiliations:** School of Health in Social Science, University of Edinburgh; School of Biomedical Sciences, University of Edinburgh

**Keywords:** arts-based, haunting, qualitative, slow death, suicide

## Abstract

Sociological research on suicide has tended to favour functionalist approaches, and quantitative methods. This paper argues for an alternative engagement - drawing on interpretive paradigms, and inspired by ‘live’ methodologies, we make an argument for a haunted sociology of suicide. This approach, informed by Avery Gordon’s haunted sociological imagination and Lauren Berlant’s concept of slow death, works between the structural realities of inequalities in suicide rates and the more (in)tangible *affects* of suicide as they are lived. These theoretical engagements are illustrated through an empirical study which used collaborative, arts-based discussion groups about suicide. The groups were held with 14 people, all affected in different ways by suicide, and attending a community-based mental health centre in a semi-rural location in Scotland, UK. A narrative-informed analysis of data generated through these groups shows the creative potential of both arts-based methodologies, and interpretive sociologies, in deepening understanding of how inequalities in rates of suicide may be experienced and made sense of. We illustrate this via two related metaphors (‘the point’ and ‘the edge’) which recurred in the data. Our analysis underlines the vital relevance of sociology to suicide studies – and the urgent need for diverse sociological engagement and action on this topic.

## Introduction: curious omissions in suicidology and the sociology of suicide

While the study of suicide (suicidology) is ostensibly a multi-disciplinary field, the position of sociology within it is limited (Wray et al. 2011; Abrutyn and Mueller 2019; Mueller et al. 2021). Research and practice relating to suicide tends to draw primarily on psychiatry and psychology, reflecting the dominance of the ‘psy’ disciplines in arenas understood to relate to ‘mental’ health (Rose 2018). The minor role sociology plays within suicidology is especially curious given the central role that suicide (via Durkheim, 1952) has played in the discipline of sociology itself. Further, it is well-established that rates of suicide are affected by, for instance, gender relations and norms, and by economic factors, pointing to the vital importance of sociological perspectives in understanding suicide (Canetto & Sakinofsky, 1998; Reeves & Stuckler, 2016). Despite this, and as charted extensively by both sociologists and critical suicide studies scholars, understandings of and research about suicide tend to favour narrow, psychological theories (Abrutyn & Mueller, 2019; Hjelmeland & Knizek, 2017; Mueller et al. 2021).

Another feature of the sociological study of suicide is a fairly entrenched emphasis on quantitative methodologies, which avoid close consideration of (in)tangible, felt, phenomenological or experiential aspects of suicide. Thus, while suicide is frequently described as ‘complex’ this rarely extends to a consideration of the multiple meanings and social practices which make up suicide. Instead, existing sociological research addressing suicide has overwhelmingly drawn on functionalist, Durkheimian traditions (Mueller et al., 2021; Wray et al., 2011 – though see Doblyte 2022 for an important exception). These approaches involve measuring rates of suicide among different populations, and carrying out correlative studies with a range of social factors, such as divorce rates, unemployment, or religiosity. Such studies generally seek to refine or enhance Durkheim’s original typology of suicides (Mueller et al., 2021; Wray et al, 2011). Additionally, such studies contribute additional knowledge about ‘risk factors’ for suicide – extending these from psychological antecedents (hopelessness, entrapment) to social factors (divorce, unemployment). The notion of ‘risk factors’ is an important one for suicide researchers, leading from the understanding that if risk factors can be identified then it is possible to alter the risk factor or – more usually – better identify and intervene with individuals who may be more ‘at risk’ of death (Marsh 2016; White and Morris 2019).

Importantly, following Durkheim, sociologists using quantitative methodologies to examine suicide generally seek to ‘fix’ or bracket off meanings in order to measure suicide as a social fact (Douglas, 1967). The dominance of Durkheimian approaches to studying suicide may reflect the centrality of Durkheim himself, as one of the ‘founding fathers’ of the discipline. However, it is also likely that the ‘scientism’ of suicidology as a whole, and a strong preference for quantitative methodologies (Hjelmeland, 2016), has meant that Durkheim’s typology and methodologies have found an easier fit than alternative, more interpretive sociological approaches to suicide. There are two significant gaps, then: firstly, sociology’s contribution to suicidology is relatively limited overall; and secondly, the theoretical and methodological tools favoured by sociologists of suicide have focused largely on replicating Durkheimian approaches, using quantitative methodologies.

A crucial way in which sociology can and should contribute more to understandings and responses to suicide may be found in more interpretive and qualitative approaches. Recent work by Mueller, Abrutyn and colleagues (2018, 2019, 2019, 2021) has begun to set out such an approach, albeit one that continues to draw inspiration from Durkheim. This work includes a ‘cultural-structural’ approach to theorising suicide, which seeks to retain key insights from Durkheim, whilst developing a more ‘robust’ incorporation of the role of culture and meaning (Abrutyn and Mueller, 2018). An empirical illustration of these theories draws on qualitative research with people living in a small, affluent community with a long-standing youth suicide problem (Abrutyn, Mueller and Osborne, 2019). In this study, Abrutyn et al. argue that explanatory social scripts about suicide among young people in this community may serve to enhance vulnerability in this cohesive and highly regulated setting.

Abrutyn and Mueller’s research offers an innovative approach to the sociology of suicide, drawing as it does on both interpretive and more functionalist theoretical resources, and testing these empirically using qualitative data. Considering the meanings or scripts assigned to suicide among different communities, and individuals within these communities, provides an alternative social shape of suicide – one less reliant on numbers of deaths or rates, and more attentive to the shape that social meanings take (Abrutyn & Mueller, 2018). This approach underlines the importance of engaging with how suicide is understood among diverse communities, since the meanings that suicide has to different groups will undoubtedly affect how they respond to suicide prevention initiatives (see also Kral, 2019; Wolsko et al. 2020). This work demonstrates how a more interpretive approach, which takes meaning and culture seriously, and is grounded in qualitative inquiry, may help to understand why rates of suicide vary across different groups; and why suicide may be patterned in other ways – such as via the methods of suicide employed, or the place of death (Chandler et al., 2022; Chandler et al., forthcoming).

While drawing inspiration from Abrutyn and Mueller’s work, we propose here an alternative interpretive approach, one which seeks to hold more space for the multivocality of suicide, whilst not abandoning a concern with structural contexts and drivers of suicide. We build upon interpretive sociologies of suicide (Douglas, 1967; Fincham et al. 2011; Langer et al. 2008; Smith, 1983), and inquire into the cultural and social contexts in which suicides occur, as an alternative way into understanding suicide variations across social groups and geographical locations. Informed by interpretive sociology, symbolic interaction and cultural studies, as well as by ‘live’ methodologies, we seek to take seriously the cultural and material diversity of suicide. In doing so, we attend carefully to the structural contexts in which such diversities play out. Inspired, in part, by the existence of social patterns of suicide – as measured in official statistics – we inquire into these, via collaborative, community-based arts-informed methodologies (Foster, 2016). Our focus in this paper is on experiences of suicide in the context of socioeconomic inequality and precarity, however, we propose that the theoretical and methodological approaches we employ have far-reaching implications for sociologists engaged not just with the study of suicide, but with a range of other ‘extreme’ experiences which are intimately connected with structural conditions. Rather than accepting common-sense explanations for relationships between disadvantage and suicide (Atkinson, 1978), we use qualitative and creative methodologies to explore the way that such relationships are accounted for and made sense of by those affected. Informed by Avery Gordon’s meditations on ‘haunting’ in the sociological imagination, our work centres intimate details and stories of ‘every-day life’ (Neal & Murji, 2015) as vital to understanding how structural conditions shape, but do not fully contain, experiences of suicide. In the analysis we discuss below, we focus on two recurring metaphors (getting to ‘the point’ and being on ‘the edge’) which participants used to explain and make sense of suicide, in ways which connected embodied feelings with structural conditions of precarity and disadvantage.

Before introducing our study and findings, in the following section we introduce two theoretical resources: Avery Gordon’s (2008) ‘haunted’ sociological imagination; and Lauren Berlant’s (2011) ‘slow death’ that we have drawn on in our ongoing examination of the relations between interpretation and meanings of suicide, and the broader social conditions that shape suicide, particularly in the context of socioeconomic disadvantage. In related ways, each of these concepts (haunting, slow death) supports a consideration of ‘delicate’ yet ‘dense’ (Gordon 2008 p. 5) connections between what was said and expressed about suicide in our research, and the broader (unequal) social and cultural contexts in which these stories were told.

### Socioeconomic inequalities: Haunting suicides and slow deaths

Avery Gordon’s (2008) *haunted* sociological imagination represents an evocative updating of a core sociological concept. Resonant with Weber’s *imaginative* approach to studying social meanings which so inspired Douglas’ (1967) early interpretive challenges to the study of suicide, and of course C Wright Mills (1959); a haunted sociological imagination attends to the ways in which power (social structure, oppression, domination) has material yet often barely glimpsed effects. In Gordon’s work, she considers the hauntings induced by the legacy of slavery in the United States, and of poverty – the workings of power. To illustrate her arguments, Gordon turns to literature, and particularly novels by Luisa Valenzuela and Toni Morrison, to demonstrate the importance of ‘unseen’ yet ‘seething’ (2008 p. 195) features of life, and history, which shape experience. With regards to slavery, for instance, Gordon argues that Toni Morrison’s writing, particularly in the novel *Beloved* ‘shows us not only that Slavery was violent and exploitative, a System, but also that even when it was supposed to be over, it could return to haunt the living’ (2008 p. 200). Haunting and ghosts offer ways of trying to imagine the connections between such structures, and how they may be articulated or felt in everyday life.

We consider haunting in light of the routine finding that deaths by suicide in the UK occur more often among communities who are socioeconomically disadvantaged, mirroring inequalities in health, wellbeing and mortality rates. Scholars of health and illness have developed wide ranging explanations for these inequalities – underlining the multiple ways in which society ‘gets under the skin’, expressed in unequal rates of obesity, drug and alcohol related harm, mental health diagnoses, cancer, heart disease and stroke (Garthwaite, Smith, Bambra, & Pearce, 2016; Mackenbach, 2012; Schrecker & Bambra, 2015). Qualitative studies have contributed further insights into how and why ‘health damaging’ practices (smoking, drug and alcohol use) may be drawn on to navigate and ‘cope’ with oppression and difficulty; emphasising practices of resistance (Smith and Anderson, 2018; Thomas et al. 2020). With regard to suicide and socioeconomic disadvantage, qualitative attention has been more limited, though contributions to a (2017) Samaritans report ‘Dying from Inequality’ by Smith (2017) and Chandler (2017, and see 2019, 2020) are important exceptions, albeit each based on meta-ethnographies of existing research. These studies underlined the centrality of emotions (particularly shame, but also anger) in understanding the ‘complexity’ of how particular socioeconomic conditions may enhance ‘risk’ of suicide.

Gordon’s ‘haunting’ offers a way to extend these initial insights into the simultaneously affective and material relations between histories - of suicides in a community, of disadvantage, and disenfranchisement – and day-to-day living. This approach pushes us to consider a relationship between socioeconomic disadvantage and suicide that goes beyond material deprivation. To consider affective and emotional aspects of this, and how these may help us to understand more of the complexity, messiness, or what Cedric Robinson referred to as the ‘nastiness’ produced by “objective’ material forces’ (1983: 442, cited by Gordon 2008 p. 193). Gordon’s ‘hauntings’ invite a closer consideration of correlations between social risk factors (such as poverty) and suicide – to inquire more deeply into how socioeconomic inequalities are felt, and how such feelings might help us better understand the directions lives lived on the edge may take.

Lauren Berlant’s (2011) concept of slow death is – like Gordon’s haunting – a set of productive ideas, or orientations, towards thinking about the ways in which experiences, emotions, or affects, can be understood as entangled with social structures, with power. Slow death (2011) refers to the ‘physical wearing out of a population’ (p. 95); an analysis which ties structural economic processes (neoliberalism, late capitalism, racism and poverty), to the smaller, embodied, intimate affects endured or resisted by those caught up in, or coping with, these processes. Slow death is uniquely well-placed to theorise health inequalities, and has been drawn on to specifically consider suicide (Marsh 2020; Mills 2018; Puar 2012). In a paper entitled, ‘Dead People Don’t Claim’, China Mills (2018) draws on slow death to think through suicides which occurred in the context of austerity policies in the UK; a regime of severe and enduring cuts to welfare benefits, overseen by a right-wing government in the wake of the 2008 global recession. Mills analyses media representations of suicides and discourse from activist groups, which tied suicide to the devastating cuts to and conditions placed on welfare benefits from 2010 onwards. Mills suggests that the ‘slow deaths’ being endured under conditions of austerity, can be understood as explaining why and how suicide may come to be understood – and enacted - as an escape.

Existing work on slow death has been largely theoretical in nature. Berlant draws on engagements with film and literature to ‘flesh out’ their theories. In China Mills’ work, slow death is extended to a consideration of suicide deaths as part of what she terms a ‘psychopolitical autopsy’, offering important resources through which to further think through the unevenly patterned affects of suicide, and how these are experienced. What is missing, though is further exploration of how far such largely theoretical mediations on slow death, and of suicide (and self-harm) can take us, particularly as applied to understanding experiences and meaning-making among those who have had close encounters with suicide – in intimate, ‘everyday’ contexts (Neale and Murji, 2015). Mills draws on newspaper and activist accounts of austerity-suicides, while Berlant, Puar, and Marsh’s articulations of ‘slow death’ provide theoretical reflections which have thus far been engaged in only a limited way in relation to empirical data. In this paper, we seek to extend these insights, in particular considering the role of resistance and active engagement with what may otherwise feel to be equally oppressive and deterministic imaginings of suicide as ‘inevitable’ for certain groups.

We think with the concept of slow death to consider the *orientations* that some bodies may have, under certain conditions. This builds on work by Saartje Tack (2019) who draws on Ahmed’s reflections on orientations, and Chandler (2019) who has drawn on Ahmed’s theoretical model to think through the orientations of bodies/selves situated in particular configurations of race, class, gender and age, considering how these relate to suicide and self-harm. Tack notes that ‘orientations are the sedimented effects of prior orientations, of history, of somatechnics, that inform the ways in which we engage with the world and those around us’ (p. 51), citing Ahmed who writes of bodies being led ‘in some directions more than others as if that direction came from within the body and explains which way it turns’ (2019 p.58). Similarly, Chandler (2019) draws on narrative interviews to consider how *certain* (white, male, economically disadvantaged) bodies may become disrupted in their orientation towards life/living, *unexpectedly* orienting them towards suicide. Below, we further develop this notion of orientations, considering how concepts of slow death and haunting can help us think through how some bodies, more than others, are drawn to ‘the edge’; and how language itself (also and always situated) constructs the ‘edge’ or ‘the point’ in relation to sociocultural and political contexts.

Both slow death and haunting address what Berlant refers to as experiences which are ‘simultaneously at an extreme and in a zone of ordinariness’ (2011 p. 96). We think that suicide is a prime example of such an experience. Simultaneously an extraordinary, disruptive event, and an experience that can be ongoing, repeated (in thought, in action, in memory) and a ‘living present’, especially for communities affected by multiple suicides. Suicides have far-reaching effects for individuals and communities; and they are also (therefore) a part of the textures that make up ‘everyday life’.

To begin to explore these issues empirically, we draw on data generated within arts-based research workshops, which explicitly invited those with direct experiences of suicide to critically engage with discourses and explanations for suicide. Our analysis considers how far the concepts of haunting and slow death may help to deepen our understanding of the complex interplay between social structures, meaning making, and actions – both life preserving and life ending. We tentatively call this approach a *haunted sociology of suicide*: an alternative sociology of suicide which works creatively with what is said about suicide, its effects and affects, and seeks to draw imaginative connections between these and the structural and cultural contexts which mutually shape both the statistics and meanings given to suicide.

## Live Methods

The data we draw on was generated as part of a study which sought to extend ‘arts-based discussion groups’ previously used to explore meanings of self-harm (Simopoulou and Chandler, forthcoming), to a study of community understandings of suicide. We wanted to explore the use of arts-based or ‘live’ methods (Back & Puwar, 2012) of research to study meanings of suicide, and how these could be situated within particular sociocultural spaces. Live methods involve a reorientation of research methods, such that the focus of inquiry is on life ‘as lived’, acknowledging researchers as irrevocably a part of this. Extending these arguments in a reflection on ‘live sociology’, Back argues that live methods are set against what he refers to as ‘dead sociology’ – a science which studies ‘fossil facts’ as inert, extractable, lifeless. Given our concern with suicide, death (slow or otherwise) and haunting, such methods seem particularly resonant.

In their 2012 manifesto for live methods, Back and Puwar set out a range of ‘aspirations’ (p. 7), including developing empirical approaches which ‘produce affects and reactions that re-invent relations to the social and environmental’ (p. 9). This call to action resonated strongly with our own frustrations with existing approaches to studying meanings of suicide, which have drawn largely on narrative or semi-structured interviews, and which increasingly generate similar, well-worn stories about suicide and – particularly - self-harm. While it is incredibly important to listen to stories about self-harm and suicide, we had begun to wonder what the purpose of gathering more of these stories might be (Tuck, 2009). We wondered what role sociology – and social science, or research – could or should have in making space to shift stories, tell different stories, to *transform* understandings (Back, 2012), rather than reproducing stories which repeated atrocities, which centred damage (Tuck, 2009). Such transformations may of course occur in even the most straightforward qualitative interview study. However, it is also increasingly recognised that drawing on visual, non-verbal, arts-based or ‘live’ methods can offer more exciting and potentially transformative opportunities to study social life – as it is lived (Back, 2012; Foster, 2016; Wilson, 2018). Crucially, such approaches may also offer a means to communicate experiences and emotion when language fails, or where our experiences exist beyond what can be spoken.

Arts-based approaches may provide a wider range of ways of telling different stories, allowing for greater space for imagination and playfulness, and are often drawn on by researchers using ‘live’ methodologies (Tarr, Gonzalez-Polledo, & Cornish, 2017; Wilson, 2018). As Victoria Foster (2019) has argued, such creative approaches can and should be central to sociological practice – particularly where we have aims towards social justice, to working collaboratively with participants, with communities, to collectively reimaging social worlds. When considering suicide, a topic often framed as ‘difficult to speak of’, we hoped that arts-based approaches would offer a means to communicate experiences and emotion when language fails or experiences exist beyond what can be spoken. Further, these approaches open up additional ways of attending to the intangible or felt aspects of meaning, and of social life – allowing Gordon’s ‘hauntings’ another way in.

### Recruitment

Across 2019, we worked closely with a community-based mental health organisation, located in Scotland, UK, to develop and run a series of ‘arts-based discussion groups’ on the topic of suicide. Initially, we planned to work with just one group at the organisation, but the response to an ‘information evening’ session about the project attracted 16 people, all keen to take part. In response, we agreed to run two separate groups for 8 people, allowing all to take part who wanted to.

The information session was an opportunity for the research team to introduce the study and the approach, and give people chance to ask questions, or share concerns. Everyone who attended took away an information sheet. After the information session, we worked with staff at the organisation to set up dates and times that would work for potential participants. Staff helped to set up the meetings, and allocated people to different groups, partly based on availability, but also working to keep the group numbers manageable.

Our first group included four men and three women, aged between 20 and 65; while the second group included four men and four women, aged between 30 and 65. All participants had different forms of experience with suicide – many had attempted or planned suicides, several had been bereaved by suicide or had friends and loved ones who attempted suicide. Some participants identified as LGBT+, all identified as White British or Scottish.

### The discussion groups

The original plan for the discussion groups was to follow that established by Chandler in an earlier study (Simopoulou and Chandler, forthcoming). This would involve 90 minute sessions, split into 30 minute blocks of discussion, art-making, and then reflection. We planned five sessions; with the first being introductory, and the following four focusing on particular themes from existing research on suicide. The aim was to provide participants with a selection of resources or materials to spark conversation, and to invite critical deliberation, discussion and questioning of common ways that suicide was talked about in research, media, policy and in charity campaigns. For instance, in a planned session on ‘talking about suicide’, we included suicide prevention campaign materials emphasising the importance of talking openly, and asking about suicide, alongside media guidelines which emphasise the importance of sharing stories about suicide only in particular, carefully restricted, ways^i^. The resources we brought included short videos, excerpts from existing research, posters or information leaflets from prominent suicide prevention charities, newspaper articles, and government policies.

In our first session, we quickly realised that adjustments would be needed. Not all participants were comfortable with art-making, the group was very lively and chatty, and the group was too large. Although we had split our group of 16 into two groups of 8, we found that 8 people was still difficult to manage and give space for all to contribute. Additionally, the first session was beset with technical challenges, as we tried to show short videos on a phone, with poor wifi and low-quality sound.

In subsequent sessions, we split the group into two smaller groups of 3-4, though sitting in the same room, and shifted to a more relaxed approach, which seemed to suit participants. Art-making materials were laid out and available throughout each 90 minute session; and each small group could move at their own pace – working through different resources and giving space for more relaxed and intimate conversations. Altogether, we held four meetings with our first group, and three meetings with our second group. For the majority of participants, feedback indicated that they had greatly appreciated and enjoyed taking part. In both cases, participants suggested they would have liked more sessions, and it was clear that for many the space we had created together during the workshops was valuable.

Both researchers were full participants in the discussion and art-making. Although clearly marked as ‘experts’, we approached the topic and sessions in a spirit of mutual curiosity and inquiry. Interested to explore different ideas and experiences relating to suicide together, and to experiment with the possibilities of sharing these via drawing and painting, as well as talking. This approach drew on Foster, who advocates for arts-based research as enabling:

‘… a diversity of experiences to be communicated in ways that disrupt ‘common sense’ understandings and act as a reminder that there are possibilities for things to be otherwise’ (2016 p. 1)

Crucially, Foster underlines the role of collaboration in these efforts; recognising the limits of attempts to ‘give voice’ to those we research with, such approaches nonetheless seek to recognise that the production of knowledge is a ‘social affair’ (Eisner, as cited by Foster 2016 p. 2).

‘A collaborative research process offers opportunities to raise critical consciousness, highlight social relations and to promote deeper understanding among participants, facilitators and audiences’ (p. 2)

The extent to which participants engaged in art-making as part of the workshops varied. We were clear throughout that participants were not obliged to contribute or take part in the arts-based aspect of the groups. Despite this, we felt that the presence of art-making throughout our meetings had important effects on the nature of participants’ engagement, and the types of conversations that were possible. This was further shaped by the collaborative ethos that we sought to cultivate among the group members. By bringing materials with us to each meeting - quotes from research papers, newspaper articles, or summaries of policy or guidance relating to suicide, we invited participants to respond to these, to question them and inquire into them. This, along with the presence of paper, paint, markers, glue and scissors, and the active engagement of some group members in using these to work with the often ‘heavy’ issues we grappled with together, lent a relaxed and curious atmosphere to our meetings.

### Analysis

Our discussions were recorded, using digital recorders placed on the tables. This resulted in two transcriptions of small group discussions for each session. In addition, both researchers wrote reflective fieldnotes after each session. The final data set included transcribed group discussions, fieldnotes, as well as artwork produced by researchers and participants.

Our approach to this data was informed by Riessman’s (2008) *Narrative-Informed Inquiry for the Human Sciences*, and in particular her argument that visual narratives and spoken narratives can be examined together. Ultimately, perhaps because the art-work was less central for many participants, the analysis developed in this particular paper focuses more closely on spoken narratives rather than images.

Analysis thus proceeded with both authors reading and re-reading transcripts, fieldnotes, and ‘reading’ the images made by participants in each session. We were attentive to common themes and issues that were raised, and to particular ways in which suicide was understood or made sense of. A key concern was the way in which stories about suicide followed particular logics, or ways of explaining; and through that, a concern with how some of these were repeated and taken up.

Through this process of reading, and writing into the data, we then began to consider theoretical resources which would deepen our analysis. This approach draws on Mazzei and Jackson’s (2013) notion of ‘plugging in’, as well as Tavory and Timmerman’s (2014) abductive analysis – where we worked between data and theory, asking questions informed by each of the other.

## Findings

In order to illustrate how the concepts of haunting (Gordon, 2008) and slow death (Berlant, 2011) can be imagined in and with our data, we introduce two recurring and related metaphors that participants drew on to share stories of suicide and suicidal crisis: the notion of ‘getting to the point’ and ‘being on or going over the edge’. Like Berlant, participants – while underlining the immediacy of a suicide ‘crisis’ - located this crisis in mundane textures and patterns of everyday life. Through our engagement with participants over several weeks of focused conversations and art-making about suicide and about their everyday lives, we were collectively able to make links between stories of immediacy and urgency, alongside deeper and longer-term structures of disadvantage.

We found Gordon’s (2008) notion of haunting helpful in further opening up a consideration of the affects of social structures and longer histories, of considering how these may shape experiences in the ‘urgent present’. To consider – as Gordon writes, drawing on Williams’ notion of structures of feeling - the ‘specific feelings, specific rhythms’ (p. 201) that are socially produced and located. For Gordon, hauntings are shared feelings, and in our data we find evidence of this – shared accounts of feeling and being on the edge, or of getting to the point where suicide becomes possible.

### Getting to the point

Participants often talked about suicide using the phrase ‘getting to the point’ – a phrase that invokes movement and travel; an orientation, perhaps – ‘getting to the point’ where suicide was a possibility, or even a distinct likelihood. In the excerpt below, Danny and Sarah Wright are discussing Danny’s experience of attempting to access help and care for deteriorating mental health problems. In Danny’s account, suicidal thoughts are framed as evidence of escalation, he had ‘got to the point’ where suicidal thoughts were so extreme that he was admitted to a psychiatric inpatient unit. The overlapping conversation, between Sarah and Danny, with Rose and Kezz joining in toward the end, reflects the dynamic nature of our workshops and discussions. Accounts of care, frustration, and incredulity at the perversity of local mental health care are co-created, and reflect a shared endeavour at seeking to expose these.

**Danny**: But, sadly, that’s where you have to get to a point, that’s where it got for me to a point where I was feeling that suicidal that I spent a night in the [name of hospital] for them to then change and - put me on different - change ma medication *completely* and put me on…

**Sarah W:** so rather than kind of working preventatively – like seeing it early on

**Danny**: they make you go -

**Sarah W**: they wait until you’re at crisis

**Danny**: yes. Absolutely. That doesnae help!

**Rose**: nothing happens til you’re in a crisis!

[…]

**Kezz**: but you’ve got to make it worse than it is, to try and get an appointment

**Danny**: no, in terms of that I didnae. I got to the, like I say, you’re at breaking point where *then* they intervene. And it didnae help, that’s what I found. Yeah, that’s no preventative, it’s more…it’s fire-fighting isn’t it? That’s what they do, it’s fire-fighting

Danny and Rose speak over each other here, agreeing strongly with Sarah’s suggestion that services wait until a crisis is already occurring before help is offered. Danny emphasises twice that not only does the ‘help’ come too late, but that the ‘help’ itself is insufficient. Reflecting a biomedical model of mental health care treatment, the first line of response – after ‘making him safe’ with inpatient ‘care’ – is to change his medication ‘completely’. Together, Rose, Danny, Kezz and Sarah discuss a recognisable pattern where services do not respond unless there is a ‘suicidal crisis’. Rose resonated with this – noting that she had also been unable to get support following the death of her partner to suicide, until she herself was ‘in crisis’, until her body had ‘got to the point’ of being in a place where her death could have occurred.

In this sense, ‘getting to the point’ could also refer explicitly to bodies *getting to* certain places where suicide became more possible. Rose spoke of ‘getting to the point’ where she ‘found’ herself in a particular location, near an infamous bridge – a known place where suicide deaths occurred.

**Rose**: And the crunch came when I found maself in [name of place] and wanting to jump off a bridge. Had no clue how I got there – I drove – but had nae recollection of driving and I thought, nah, I need to get help.

In Rose’s account, the point (or ‘crunch’) where she ‘found’ herself near the bridge, was a moment of realisation for herself, prompting a visit to a GP, and leading to her eventually beginning to attend the community-based mental health service where we held our workshops. The need to get to such a ‘point’ before seeking help (or being granted it) was situated in a broader context of significant levels of stigma and shame in relation to mental health problems, as well as negative experiences with previous ‘care’. Rose shared that she had previously spent a year as an inpatient in a psychiatric hospital, a time she described as ‘the worst fucking year of my life’. The ‘fear’ of being returned to hospital was described as a significant barrier in terms of seeking help for ongoing distress. Rose’s account emphasised that the reaching of a ‘point’ where death was possible was necessary before she could get help.

In our discussions, suicide was framed as something that one ‘gets to the point’ of; and seeking help for distress something that was promoted or permitted by ‘getting to that ‘point’. However, participants also emphasised that despite such a ‘point’ being one of crisis – an extreme point, something overwhelming or unbearable – the professional response to such a crisis was framed as often insufficient: it does not help, because it is too late – crisis has already been reached. It misses the point.

### Being on the edge

As well as ‘getting to the point’, participants spoke of being on or going ‘over the edge’ as a way of explaining and narrating their experiences with suicide. This turn of phrase contained similar elements of urgency and precarity, underlining the contingent nature of suicide and self-harm. When one dwells on the edge it is more possible to fall off. In this section, we introduce data which drew on the notion of being on, or going over, the edge, before turning to a broader consideration of how we might make sense of participants’ journeys to ‘the edge’, or ‘the point’, as a way of enrolling structural considerations in our interpretation of intimate tales of distress.

Sarah B attended our first workshop series. Here she relates her own experience of ‘crisis’, of being ‘tipped over the edge’ by the loss of a bus pass.

I was a mess. I’d missed work. I’d lost my bus pass, and it tipped me over the edge, just that one thing, and I was close to [hospital] and I walked in and - I’ve got a lot of pride - but that day I was a mess; and I walked in and I’d not had a cigarette in like two weeks cause I was tryin’ to quit and go all healthy and that and I just snapped … I was at the front desk and I was like ‘you have to lock me away cause I’m…’ [Jane interjects – ‘gonna hurt yoursel”] like literally begging and then it was A&E and they were just like ‘oh just sit down’ you know what I mean, and nothing - and then I spoke to a woman and then she referred me to my doctor and that was that **Sarah B**.

Crucially the cumulative building up of events, and the ‘crisis’ where Sarah B finds herself ‘over the edge’ and strongly desiring death, is responded to *without* a similar sense of urgency by the healthcare professionals she consults. Sarah B’s moment of crisis, of ‘snapping’ and ‘going over the edge’ is narrated as being responded to as if a naughty child – ‘oh just sit down’. We get a sense here (and Sarah B’s story is not unique in our data by any means) that the urgency of the crisis is also a normal part of life in a hospital. Another person in distress who wants to die. The potent urgency - ‘begging’ to be locked away - is referred back home. Matthew related an eerily similar story – of being sent home from A&E just hours after admission following a suicide attempt. His family expecting an admission, expecting some kind of intervention. Matthew told us – smiling wryly - that instead he was discharged home with the promise of a follow up phone call, which 6 months later was still forthcoming.

As with the stories we heard of ‘getting to the point’, the response to those who are on or over ‘the edge’ was narrated as not recognising the urgency of that place (edge, point). The crisis was not attended to. This lack of institutional response is of course also shaped by conditions of scarcity – including, but not restricted to, long term under-funding of mental health services, further cuts to the National Health Service under the UK government’s austerity policies, and privatisation by stealth (Tyler & Slater, 2018).

At the same time, the cumulative disadvantages that *lead* someone towards the edge are *also* left unaddressed and silent. However, within our workshops, participants explicitly named these disadvantages, tying them to the experience of being ‘on’ or ‘over’ the edge. The week after Sarah B had recounted her tale of losing her bus pass, and being tipped over the edge, Pete, Jane and Marshall returned to this metaphor as they discussed challenges that Marshall was facing in accessing welfare benefits. Marshall was often very quiet in the groups, focused on listening to others, whilst drawing striking pictures using coloured paper and marker pens. That week, she recounted in detail being told she was ineligible for benefits, and her fears about providing for her family. Jane offered practical help about how Marshall might better navigate the complex and frustrating system, though Marshall herself appeared unconvinced and deflated:

**JaneW**: so go to the job centre, erm, go to your doctor get a sick line

**Marshall**: I’ve got a sick line

**JaneW**: ok, you put that to the job centre and then you’ll get universal credit but then they’ll be a letter like the PIP one – the big form - and get [service provider] to help you fill it out and then send it away and then you’ll get another appointment like the PIP appointment, but it’s not, it’s universal credit appointment and then they’ll put you on the ESA

**Marshall**: I just feel like there’s no point

There was a pause here, and then Marshall turned to Sarah Wright and said ‘Anyway, that’s … sorry – I digress’. Sarah reassured Marshall that this exchange was ‘not irrelevant’.

We returned often to this moment in our analysis, particularly given the conversation then developed further, with Jane and Pete seeking to further reassure Marshall about how normal her feelings of helplessness were:

**Pete**: I’m the same, it’s like when you’re going through this all you want is for somebody to pick you up, and say ‘I’ll deal with it’

**JaneW**: ‘cause you can’t cope with it

**Pete**: you can’t, you can’t process it and it could be like, it could be like the girl said last week, I couldn’t find my bus pass and it tipped me over the edge [breaks down laughing here]

**Marshall**: yeah

**Pete**: and it could get at that level where it could be

**JaneW:** Anything

**Pete**: Anything. Yeah. It could be like the slightest thing and then you’re just

**JaneW**: yeah

**Pete**: upset, that’s it

**JaneW**: it’s the straw that breaks the camel’s back

In this exchange, Pete and JaneW provide a ‘playful’ (Foster, 2019) illustration of Berlant’s slow death. Drawing on metaphors of ‘the edge’ and ‘the straw that breaks the camel’s back’, they point to the importance of cumulative set-backs and challenges in orienting towards crisis, towards death. While this discussion was certainly one that faced the challenges of suicide, and associated difficulty, head on, we want to draw attention also to the role of shared humour – or perhaps incredulity – in this exchange and the others above. Participants across the group told these stories not only as ‘atrocity tales’ or as ‘damage centred’ narratives (Tuck, 2009). There were notes of resistance – evident through the sharing of absurd or darkly humorous accounts, which sought to underline the often ludicrous way in which mental health crises were responded to (or not), set against exhortations from public mental health and suicide prevention charities that those in distress should ‘seek help’ and ‘reach out’ (see also Chandler, 2021).

That week, Marshall’s^ii^ drawing featured a repeated rendering of the ‘scream’ face (which could be read as reflecting either or both Edvard Munch’s ‘The Scream’ or the ‘Scream’ mask used in the comedy-horror film series of the same name) in black marker, on a white piece of card. Threaded through the repeated screaming faces were a number that Marshall told us was special and protective to her, as well as the words: die, loser, help, voices, checking, fear.

## Discussion: towards a haunted sociology of suicide

### Moving towards death

‘Could it be that analysing hauntings might lead to a more complex understanding of the generative structures and moving parts of historically embedded social formations in a way that avoids the twin pitfalls of subjectivism and positivism?’ (Gordon, 2008 p. 19)

Gordon’s writing on hauntings and the sociological imagination have helped us to consider the complex, (in)tangible ways in which suicide – as a meaningful social practice – is shaped by context. In our analysis, we sought to move beyond a linear and simplistic rendering of social disadvantage and precarity as correlated with and productive of suicide. Instead, drawing on the lively discussions in our arts workshops, we listened and watched for the subtle ways that structures might ‘haunt’ what was said about suicide.

In a closely resonant way, Lauren Berlant’s conceptualisation of slow death also supports a more nuanced examination of how social structures and cultural meanings become intimately entangled with habits, practices, embodied ways of ‘going on’ with life. Such an approach works against a more usual tendency in suicide studies to either bracket off agency almost entirely (as per Durkheim’s original arguments), or to render agency in ways Berlant might frame as ‘inflated’.

‘Through the space opened up by slow death … I seek to recast some taxonomies of causality, subjectivity, and life-making embedded in normative notions of agency […] we need to think about agency and personhood not only in inflated terms but also as an activity exercised within spaces of ordinariness’ (Berlant, 2011 p. 99)

For both Berlant and Gordon, ordinary ‘everyday’ spaces are crucial sites where structural and historical damage may slowly accumulate and – potentially – be resisted. In our conversations with participants across the workshops, numerous stories were told of such ‘everyday damage’. Through the metaphors of ‘the point’ and ‘the edge’, participants created deeply affective accounts of the subtle ways in which suicide might become more possible when reaching such ‘points’ or being pushed towards a real, or symbolic, edge.

The haunted sociology of suicide we propose here provides a way of thinking between what people say, what suicide evokes, and the social – and cultural – contexts in which stories are told, lives are lived and in which deaths occur. It is a way of considering the intangible ways in which suicides affect and are affecting. At the same time, it allows us to consider some of the complex and unseen ways in which power operates on lives and deaths. Suicide haunts the accounts of our participants, as a potential future for those who get to ‘the point’ or who end up on, or over ‘the edge’.

Suicide in this imagining is both a crisis and not a crisis. As with Berlant’s slow death, the stories told by those dwelling with suicide contain urgency but also articulate the slow, grinding down of unfeeling structures, providing a more complicating understanding of how economic insecurity is lived, and how it might produce feelings that lead one to an edge, or to the point, where suicide becomes a possibility (and see Mills 2018).

Future sociological research on suicide might build further on our haunted sociology of suicide in a number of ways. We have demonstrated here, with a focus on socioeconomic disadvantage, the productive connections that can be drawn between qualitative accounts (particularly as generated in arts-based research) and the structural ‘shape’ of suicide. Suicide is patterned in other ways – and a haunted sociology of suicide might also help to deepen existing analyses of how gender, race/ethnicity, and sexual orientation relate to suicide (Chandler 2019; Jaworski 2014; Marzetti et al. 2022.). Further, a haunted sociology of suicide is highly amenable to a more intersectional approach (Collins, 2019) emphasising the need for sociologically informed, qualitatively driven studies of suicide which attend to the complex ways in which meanings and actions are shaped by intersecting structural factors.

A haunted sociology of suicide underlines the urgent need to engage closely with the meanings that suicide has for diverse groups, expanding our sociological imagination to consider how these meanings are entangled with – and indeed may shape – the social patterns of suicide. Further, this approach entails a consideration of suicide in relation to space and time – another often neglected aspect in suicide research (Chandler et al., forthcoming; Stevenson 2016), inviting further inquiries. Haunting – and slow death – each guide our attention to histories and their ongoing impact in the everyday, including how such histories can be emplaced – within particular geographies and material settings.

### Resistance and the limits of hope

In closing this paper, we want to briefly attend to some of the threads of resistance that can often become absent in theoretical uses of slow death, and which our empirical work, and engagement with ‘live’ methods sought to make space for. In our work with participants, we deliberately introduced materials which provoked disagreement, and which encouraged dissent. The tenor and tone of the discussions we focus on above reflect something of the ‘lively’ space that we created together in our workshops. The inhumane responses of local services to the ‘crises’ that participants described were mocked, held up as ridiculous. We laughed together. The situation was not accepted. Participants worked together to resist and work around the structural conditions which shaped the ‘edges’ to which some were drawn. In side conversations, advice about benefits, participants created a place of recognition and solidarity. Our workshops were certainly not the only factor here. Rather, the workshops and the space we held were made possible by the existence of the community centre and its dedicated staff. Staff and community members who worked together to support each other through ‘crises’ and to offer more everyday comforts – meals, coffee, cake and advice.

Our research was carried out just prior to Covid-19. During the subsequent months and years of lockdowns, home working, and attendant struggles, we maintained limited contact with the community-based mental health centre where we held our workshops. Whilst we developed the analysis we present here, the community centre itself struggled to survive. Eventually, the centre was closed, with service users offered – by way of replacement – one-to-one counselling. We continue to reflect, with rage and exhaustion, on the implications of this shift, and on the losses and limits it entails.

## Data Availability

Due to the sensitivity of the data, and small sample size, data from this pilot study are not publicly available.

## Footnotes

i In the UK, the suicide prevention charity Samaritans has well-publicised and widely used media guidelines – https://www.samaritans.org/about-samaritans/media-guidelines/ (accessed 9^th^ March 2023).

ii Each participant who made art as part of the group chose what to do with the art itself. They could throw away the art, allow the researchers to take images, but take the art home with them, or ‘donate’ the art to the project. In this case, Marshall wanted us to keep the art, but did not explicitly give permission for it to be shown. We had planned a longer term engagement at the community centre, where we would have developed a public exhibition or similar, but this was derailed by Covid-19.
